# Relations of Pulse Wave Velocity to Waist Circumference Independent of Hip Circumference

**DOI:** 10.4178/epih/e2010004

**Published:** 2010-05-03

**Authors:** Min Jung Ko, Mi Kyung Kim, Jinho Shin, Bo Youl Choi

**Affiliations:** 1 Department of Preventive Medicine, College of Medicine, Hanyang University, Seoul, Korea.; 2 Health Insurance Policy Research Institute, National Health Insurance Company, Seoul, Korea.; 3 Department of Internal Medicine, College of Medicine, Hanyang University, Seoul, Korea.

**Keywords:** Abdominal obesity, Waist circumference, Hip circumference, Pulse wave velocity, Residual method

## Abstract

**OBJECTIVES:**

Little is known about the effect of waist circumference (WC) on brachial artery pulse wave velocity (baPWV) independent of hip circumference (HC). Therefore, this study aimed to dissociate specific effect of WC on baPWV independent of HC.

**METHODS:**

Of 1,053 rural residents (2004-2005), 777 subjects with no known history of coronary artery diseases or diabetes mellitus over 40 yr were included. To reduce collinearity, we assessed the independent effect of WC with HC on PWV by residual method (WC [RM]).

**RESULTS:**

In women, most correlation coefficients were significant between measures of abdominal obesity and baPWV, with the highest (0.32) in waist to hip ratio (WHR), whereas no significance was found in men. All mean values of baPWV among the abdominally obese were higher than those of normal group in women, which were in the order of WHR, WC (RM), and WC. Adjusted OR with 95% CI for baPWV was significantly elevated by increase of WC (RM) upto 4.8 (95% CI: 2.1-11.2), and as 4.3 by WHR (95% CI: 1.6-11.4).

**CONCLUSION:**

Considering the difficulty in biologically interpreting WHR, WC (RM) may be a useful indicator of abdominal obesity among females in that it reflects the risk of pulse wave velocity.

## INTRODUCTION

Elevated arterial stiffness increases the risk of cardiovascular disease (CVD), including myocardial infarction, heart failure, stroke, dementia, and renal disease, and finally leads to the elevated total mortality [1, 2]. Pulse wave velocity (PWV) is widely used as an index of arterial stiffness [3] and a marker of vascular damage [4].

The stronger association between abdominal obesity and pulse wave velocity than peripheral obesity might be explained by the effect of insulin resistance [5-7]. There is no doubt that dual-energy X-ray absorptiometry (DXA) or computer tomography (CT) can more accurately quantify the body fat depot than anthropometric measurements. However, practical issues in population studies [8] rendered anthropometric index further used for characterizing abdominal obesity. The waist circumference (WC) alone is a preferred indicator for abdominal obesity mainly because of 1) its close connection with visceral fat [9-11], 2) the stronger association with CVD [12] or type 2 diabetes [13], and 3) the simplicity of measurement and the ease of interpretation [14]. It, nevertheless, has a limitation of not incorporating body composition. Instead, waist to hip ratio (WHR) reflects the body shape and lower trunk adiposity in some degree [15]. Again, WHR requires a caution for interpretation; a high WHR indicates a smaller hip circumference (HC) as well as an excess of visceral fat [16, 17].

When the WC is considered, a larger HC has been shown to be protective for diabetes [18], myocardial infarction and mortality of CVD [19] in a different ethnic group [20]. It might be explained by the mechanism that interplays in HC and muscle mass, femoral mass and skeletal frame lowers the risk of CVD determinants [21, 22]. A line of studies have consistently reported the independent and often opposite association between WC and HC in the levels of glucose and lipid, diabetes, and CVD [18, 19, 23]. But no previous studies have elucidated the specific effect of WC on PWV, to our best knowledge.

Therefore, the present study aims to dissociate the WC effect independent of HC to reduce the collinearity between WC and HC, and determine the relationship with PWV.

## MATERIALS AND METHODS

The study place, Yangpyeong County consists mainly of farmlands where over 50% of adult residents are farmers. To recruit study population, we first advertised targeting local leaders of each district and the leaders, in turn, motivated residents by word of a mouth. As a result, a total of 1,053 subjects aged over 40 yr (male: 408, female: 645) participated in the health survey in Yangpyeong County in 2004-2005. Of these we excluded those with known history of any of the following: angina, myocardial infarction, stroke, or diabetes mellitus (163 cases), with any missing or extreme value (±3 standard deviation; SD) in brachial artery pulse wave velocity (baPWV) (81 cases), and with any missing value across major variables (32 cases). The final sample, thus, included 777 subjects (288 men; 489 women).

All anthropometries and blood pressure (BP) were measured by well-trained personnel. Height was measured while wearing a standard gown to the nearest 0.1 cm using a stadiometer. Weight was determined to the nearest 0.1 kg on a metric weight scale. Waist circumference was measured at the smallest horizontal trunk circumference and hip girth was measured at the largest horizontal circumference around the hip and buttocks, with non-stretching fiberglass or metal tapes (SECA, Hamburg, Germany), to the nearest 0.1 cm. WHR was, then, calculated as waist circumference divided by hip circumference. BP was measured with a standard mercury sphygmomanometer using the first and fifth Korotkoff sounds, to the nearest 2 mmHg in a sitting position at least 5 min (Baumanometer). Pack-year was calculated by multiplying the number of packs of cigarettes smoked per day by the number of years the person has smoked. Arterial stiffness was measured by brachial artery pulse-wave velocity (baPWV). The baPWV was defined as the distance between right brachial site and each ankle site divided by the pulse wave transmit time (PTT) from ascending point of right brachial pulse volume recording (PVR) to ascending point of each ankle PVR (Colin VP-1000; Colin Co., Ltd., Komaki, Japan) [24]. Pearson's correlation coefficient of reproducibility was r=0.961, and the interobserver coefficient of variance was 5.4% (n=17) when measured by a staff, residing in this study area [25]. In the present study, mean right and left baPWV value was calculated during analysis.

The Institutional Review Board of Hanyang University Medical Center approved this present study and all participants signed the written informed consent form approved by the Institutional Review Board of Hanyang University Medical Center.

### Criteria

Hypertension was defined as a mean BP ≥140 mmHg systolic or ≥90 mmHg diastolic, or current use of antihypertension medication. Overweight was determined as a body mass index of 25 or more. Abdominal obesity by WC was defined as WC ≥90 cm in male and WC ≥80 cm in female, according to Asian's criteria [8]. The criterion of abdominal obesity of high WHR was defined as WHR ≥0.9 in male and WHR ≥0.85 in female [26]. The cutoff value of normal baPWV was determined as a value at or less than mean +1 standard deviation (SD) among those whose BP level of below 140/90 mmHg and no known history of hypertension medication according to age group (<55 yr, ≥55 yr).

### Statistical methods

Values were expressed as mean values with standard deviations unless otherwise specified. The distribution was normalized through a natural log transformation (baPWV) or a square-root transformation (WHR) during analysis. The mean or prevalence difference by gender was examined by t-test and chi-square test, respectively. Major covariates in this present study were age, systolic BP (SBP), heart rate, pack-year, and BMI. Pearson correlation coefficients were estimated to assess the association between each of obesity indexes and baPWV, and partial Pearson correlation was assessed after adjusted for covariates. General linear model adjusting for major covariates was employed to compare the mean level of baPWV according to abdominal obesity index. The index of WC independent of HC was estimated based on the residual method [23]. Residuals of WC and HC were first estimated based on the difference between observed and predicted values of BMI and age; these ones were included in the general linear model as independent variables along with age, BMI, and residuals of the other circumference. The cut-off value of abdominal obesity according to WC independent of HC based on the residual method (WC [RM]) was defined as a value at or above the 90th percentile by sex. Logistic regression was performed to examine the degree of the association of baPWV across the quintiles of WC, WHR and WC (RM). The adjusted odds ratio (OR) for major covariates is presented according to each quintile group. Further adjustment for the residual of HC was done in the analysis of WC (RM) and baPWV. The linear trends across quintile categories of WC, WHR and WC (RM) were estimated by treating the categorized variables as continuous variables assigned with the median value within the category in the logistic regression models. All analyses were conducted using SPSS software (version 12.0, SPSS Inc., Chicago, IL, USA), and p-value<0.05 was considered statistically significant.

## RESULT

The mean age of the subjects was 60.9 yr with a SD of 10.3 yr (Male, 62.2±10.0; Female, 60.1±10.4); 62.9% were female (Table 1). Mean values of BP and baPWV were higher in males, whereas mean level or prevalence in obesity domain were remarkably higher in females (men: 42.4%, women: 82.8%). Likewise, all prevalence of overweight and abdominal obesity was predominantly higher in females.

**Figure 1:**
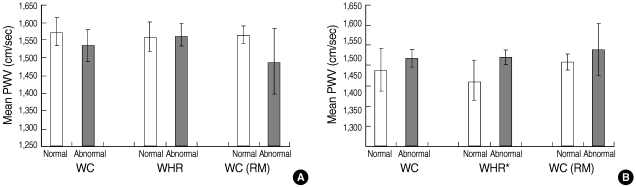
Mean pulse wave velocity (PWV) with 95% confidence interval by obesity groups defined by waist circumference (WC), waist to hip ratio (WHR), and waist circumference independent of hip circumference based on the residual method (WC [RM]) (A: male, B: female).

**Figure 2:**
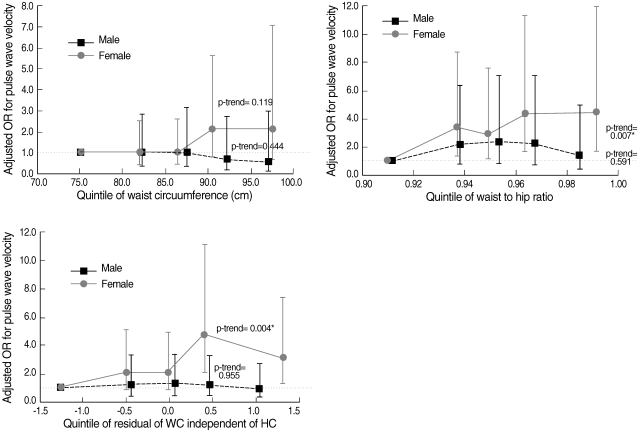
Adjusted odds ratios^*^ with 95% confidence interval for high pulse wave velocity (PWV) by the quintiles of waist circumference (WC), waist to hip ratio (WHR), and waist circumference independent of hip circumference based on residual method (WC [RM])^†^. ^*^Adjusted for age, systolic blood pressure, heart rate, pack-year, hypertension, and BMI; ^†^Further adjusted for the residual of hip circumference. OR: odds ratio.

In females, most correlations between abdominal obesity index and PWV were significant with a highest level of 0.32. However, no significant correlation was found in men (Table 2). In both sexes, the partial correlation coefficient between WHR and baPWV was the highest (male, 0.03; female, 0.16), while the difference between WHR and WC or WC (RM) did not greatly differ. In addition, the level of partial correlation between either WC or WC (RM) and baPWV was the same (male, -0.04; female, 0.13). When comparing the mean values of baPWV according to each index for abdominal obesity, no significant difference was found in all three obesity indices in males, which is shown in Figure 1. In male, the mean levels of baPWV (cm/sec) belonging to abnormal group either by WC or WC (RM) were even lower than those of normal group; the average of normal and abnormal by WC was 1,573.3±40.8 and 1,535.4±47.2, respectively, and by WC (RM) was 1,560.9±25.8 and 1,537.4±77.8, in order. No difference was observed between the categories of WHR. By contrast, all mean values of baPWV in abdominally obese groups were higher than those in normal ones in females. The difference was the highest in WHR group (normal, 1,461.8±43.3; abnormal, 1,520.7±19.7, p<0.05) and then WC (RM) (normal, 1,506.8±18.8; abnormal, 1,566.7±58.2) and WC alone (normal, 1,489.4±52.2; abnormal, 1,517.0±20.3), in order; it was only significant in WHR. To assess any changed pattern in the risk of baPWV by the increase of each obesity index, the adjusted OR of quintiles was estimated and compared in Figure 2. It depicts little difference in the risk of baPWV by the increase of all indexes across the quintile categories in males. In contrast, the increasing trend was consistent and prominent in females in all indexes. On the basis of first quintile in WHR, the OR (95% confidence interval, CI) in each quintile from second to fifth significantly increased as following (p for trend=0.007); 1) 3.35 (1.29-8.73), 2) 2.89 (1.09-7.65), 3) 4.28 (1.61-11.40), 4) 4.45 (1.65 -12.00). In particular, the increasing pattern was the most significant between WC (RM) and the risk of baPWV, after additionally adjusting for the residual of HC (p for trend=0.004); 1) 2.10 (0.87-5.08), 2) 2.02 (0.82-5.00), 3) 4.79 (2.05-11.16), 4) 3.09 (1.28-7.43).

## DISCUSSION

This study suggests that WC independent of HC based on the residual method is a useful indicator of the relationship between abdominal obesity and the risk of pulse wave velocity in females.

A line of studies have consistently documented the increased risk of arterial stiffness due to abdominal obesity. Using DXA or CT, several cross sectional studies have explored the relationship between trunk fat and elevated arterial stiffness than other peripheral fat in elderly [27, 28] or a younger population [29]. Health, Aging, and Body Composition, a prospective study with a mean age of 74 yr, demonstrated the strongest association between visceral fat and higher arterial stiffness determined by carotid-femoral PWV [6]. In addition, a largerWC reported to be a risk factor for aortic stiffness mainly in elderly women from the Cardiovascular Health Study [30]. Also in our finding, the correlation between abdominal indices based on anthropometric measurements and PWV was stronger after adjusting for BMI, which more delineated the effect of abdominal obesity. However, this relationship is less clear when using the anthropometric indices than based on the body fat depot [31]. Along with the limitation of anthropometry indices, HC might decrease the strength of association between WC and arterial stiffness. In this study, WC alone did not show any significant relationship with baPWV. Instead, the increased WC (RM) or WHR reflected the elevated risk of baPWV, both of which considered HC. Accordingly, it clearly implicates that using WC alone may underestimate or even null the waist-specific effect on arterial stiffness. It also emphasizes the consideration of HC in the obesity-related risk, against the growing movement to disregard the HC due to inaccuracy of its measurement [19].

Although the mechanism underlying the protective role of a larger HC in most CVD risk factors or diseases remains inconclusive, several candidates have been suggested as follows [22]. First of all, a larger HC is directly associated with greater muscle mass whose primary function is glucose disposal [32]. The negative relationship between lean mass in the legs and glucose levels has also been reported from the Hoorn Study among 556 participants aged 60-87 yr [28]. Secondly, the HC may also reflect gluteal fat mass accumulation. Fat tissue in the femoral region could buffer the free fatty acid levels by sinking it in blood, which protects the liver from high exposure. This consequently results in the production of a relatively lower level of low-density lipoprotein cholesterol and the activation of insulin clearance [33]. Based on possibilities mentioned above, several studies have demonstrated the independent and often opposite association between WC and HC for metabolic risk factors by cross-sectional studies [23], and in the risk of type 2 diabetes [18], and mortality [19] by prospective studies.

Meanwhile, WHR also showed significantly positive relationship with the risk of baPWV as well as WC (RM). Several reasons may account for this finding. Although the WC has been widely accepted indicator for abdominal obesity in western countries, whether the predictive power of WC may be extrapolated into Asian countries still remains controversial [9, 34, 35]. Instead, several population studies in Asian countries such as Japan [36] and Iran [35] have advocated WHR rather than WC for determining abdominal obesity. Another possibility for significant association with WHR may be explained by the fact that participants of this study were composed of fairly older group with a mean age of 60.9 yr. According to Health Professionals Study, a 3-yr prospective study among 29,122 US men aged 40-75 yr, WHR was more strongly associated with coronary heart disease among those aged 65 and older [37], whereas WC was more closely related with CVD risk factors mainly among younger white populations [38]. Nevertheless, one should again consider that WHR is difficult to interpret biologically for the nature of ratio. Moreover, the inverse term of HC included in WHR may overestimate the protective effect of WHR than as it is.

To dissociate WC specific effect independent of HC, most studies have mainly performed the multivariate regression analysis by using risk factors as the dependent variables and WC, HC, and other variables as the independent variables [16, 18, 21, 23, 28]. However, it may lead to overcorrected models for the collinearity between WC and HC, which consequently yields to marginal or no effect of WC on risk factors.

Another approach has employed the residual method to separate specific effects among fairly correlated variables, mainly used in nutritional epidemiology. Furthermore, Seidell, et al. [23] applied residual method in exploring the relationship between WC and HC with CVD risk factors, from the cross-sectional study among 695 middle-aged residents in Canada. They reported that WC and HC reflected different aspects of body composition, and, therefore, resulted in independent and often opposite effects on CVD risk factors. From this point of view, we extended the residual method into the field of arterial stiffness.

In addition, there was a remarkable gender difference in most results presented here. Not only was no significant finding found in males, but the relationship of abdominal obesity with baPWV was also the opposite between men and women. In general, a weaker association in this field among males has been reported in a prospective study among the elderly [30], mainly due to overestimate the length of carotid-femoral segment, which exaggerates the measurement of aortic PWV. However, the reason is still inconclusive; because most studies were restricted to men or women only [31], or the analyses were not made by gender [6, 29]. Meanwhile, a prospective study reported the significant relationship between a larger WC and increased mortality in never smoker males, whereas no results were found among ex or current smokers [39]. Considering the controversy over the categorization of exsmoker, we, thus, performed an adjustment for pack-year, along with other covariates in every analysis. Nevertheless, no difference was reported between unadjusted and adjusted analysis.

Besides, to evaluate the effect of potential covariates on each lipid indices, this study included total cholesterol, High Density Lipoprotein (HDL), Low Density Lipoprotein (LDL) and triglycerides as covariates. However no significant results were found (data were not shown).

To interpret the findings of this study, certain limitations should be noted. First, although this is a population based study, it has a cross-sectional design. Second, the study population had a substantial prevalence of abdominal obesity. However, it may not be exceptional considering for following reasons. We, first, applied the criteria for Asians [8], which is stricter than that of conventional one. Moreover, the Third Korea National Health and Nutrition Examination Survey in 2005, also reported high prevalence of abdominal obesity in over 60 yr and older (female: 66.7%, male: 33.3%), which is more higher among rural residents [40]. Third, we determined abdominal obesity with the use of simple anthropometric indices and no direct measures of body fat or muscle composition were provided. Instead, we compared several measured anthropometries rather than single self-reported one. Finally, the ba-PWV was employed to determine the level of arterial stiffness, which may be influenced by the peripheral measures [21]. Nevertheless, a growing number of studies, mostly in Japan [24], have demonstrated the validity of high predictability for cardiovascular deaths and events as well as reproducibility. Furthermore, it may be more suitable to screen in a large population as it is relatively simple in measuring PWV and cheap.

Despite these limitations, the present study should deserve a consideration as it beefs up the analytical methodology in assessing the effect of WC on vascular health though direct application of residual method.

In conclusion, WC independent of HC based on the residual method may be a useful indicator for pulse wave velocity in women, but not in men. Dissociated effect of WC may reflect the risk of arterial stiffness; otherwise the effect may be either underestimated or nullified by WC alone, due to the protective effect from a larger HC. Hence, further studies in determining the relationship between abdominal obesity and the risk of arterial stiffness should consider the hip circumference to estimate more accurately.

## Figures and Tables

**Table 1:**
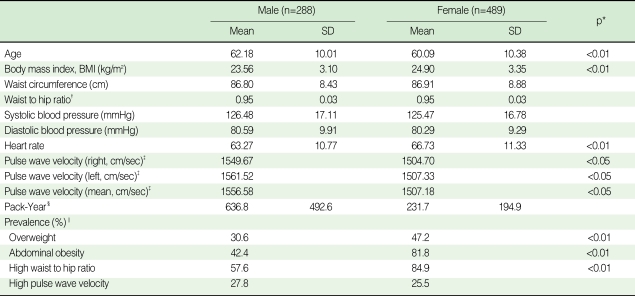
Anthropometry and pulse wave velocity related variables

^*^p<0.05, p<0.01 by either t-test or chi-square test; ^†^Square-root transformation; ^‡^Express antilog values after log transformation; ^§^Pack-Year: Number of packs per day X smoking year, ^∥^Overweight: Body mass index ≥25 kg/m^2^, Abdominal obesity: male ≥90 cm, female ≥80 cm, High waist to hip ratio: male ≥0.9, female ≥0.85, High pulse wave velocity: at or above mean+1 standard deviation by sex and age group (<55 yr, ≥55 yr).

**Table 2:**
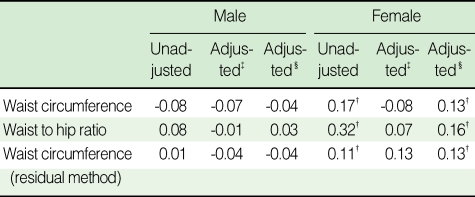
Pearson and partial correlation between obesity indices and pulse wave velocity

^*^p<0.05; ^†^p<0.01; ^‡^Adjusting for age, systolic blood pressure, heart rate, and pack-year; ^§^Adjusting for age, systolic blood pressure, heart rate, pack-year and body mass index.
